# Complications in cochlear implant surgery

**Published:** 2015

**Authors:** DC Gheorghe, A Zamfir-Chiru-Anton

**Affiliations:** * “M.S. Curie” Hospital, Bucharest, Romania; “Carol Davila” University of Medicine and Pharmacy Bucharest, Romania; ** “G. Alexandrescu” Emergency Hospital for Children, Bucharest, Romania

**Keywords:** cochlear implant, complications

## Abstract

For the last 6 years, cochlear implantation has become a standard practice in our department. The number of patients rose from 5 to 21/ year. Using multiple types of cochlear implants and indicating the surgery also to malformed inner ears led to the encounter of some complications.

**Objective:** to present the surgical complications from our department.

**Material:** all the patients admitted and operated in our clinic have been reviewed.

**Results:** 9 complications (8,86%) have occurred: the impossibility of establishing a reliable cochleostomy (due to ossification), air in the cochlea through lack of sealing of the cochleostomy (exteriorization of the electrode array), cochlear implant postoperative migration from its bed, weak hearing discrimination due to “double electrodes” in the scala tympani, gusher.

**Conclusions:** cochlear implanting needs to respect the technical steps of the surgery and the best technical/ tactical solution has to be found to whatever complications arise in complex or malformed cases!

## Introduction

Cochlear implants have developed rapidly in the last 20 years, due to technological progress of the electronic and computing devices and to continually adapting speech coding strategy to match the auditory nerve demanded stimulation [**1**]. Consequently, the treatment for hearing impaired people recorded a large success [**2**-**4**]. A lot of progress has also been recorded in public health systems supporting deafness and financing the large expenses occurring with implantable devices. Neonatal hearing screening added to the amount of cochlear implants needed in daily practice [**5**]. 

The techniques described in literature from the beginning of the cochlear implant surgery were different according to authors and manufacturer of the device [**6**,**7**]. Specialty papers described issues regarding flap infection or survival problems, wrong positioning of the electrode array in the inner ear, facial paralysis, postoperative vestibular dysfunction and mastoiditis [**8**-**11**].

Cochlear implant surgery has been performed in our department from 2009, with 79 cases recorded in our statistics. The rate and type of complications in our service seemed somehow different from the ones described in the specialty papers and that is why we brought up un update derived from our experience, with concern to this kind of surgery. 

## Material, method

All the patients admitted in our department and operated for cochlear implants have been reviewed and the complications found were recorded in the present study. **Table 1** presents the kind of complications that were encountered in our cochlear implant surgery. Clinical and imagistic data has been recorded by our team, analyzing the factors contributing to the complications described. 

**Table 1:** Complications after cochlear implant surgery

COMPLICATION	Age of the patient	Number of cases
Cochlear implant migration from its osseous bed	17y, 6y	2
Electrode array dislodgement from the cochlea	6y; 15y	2
Ossification with poor patency of the cochlea	2y	1
Gusher intraoperatively	3y;8y	2
Normal hearing thresholds with low auditory discrimination	9y	1
Neurological symptoms after cochlear implantation	2y	1

## Results

79 cases of cochlear implants were performed in our department between 2009 and 2015. Nine complications were recorded (8,86%). We reviewed them and discussed possible mechanisms and eventual prevention methods.

One case presented with cochlear implant migration from its osseous bed. The symptoms were obvious at 6 months postoperatively with the patient having local pain due to skin compression between the sound processor and the implanted device. She was tempted not to use the cochlear implant consequently. The palpation of the retroauricular region brought enough data for diagnosis. The surgical review of the implant bed without electrode array mobilization resulted in symptom relief. It was noted that the electrode array was a perimodiolar type. 

2 cases with dislodgement of the electrode array from the cochlea were recorded. The first was a consequence of revision surgery for migrated implanted device from its osseous bed (patient was referred from another ENT department). The electrode array of the implant was a straight one. The minimal mobilization of the implant but without mastoid cavity exploration was performed. Bad hearing postoperative thresholds demanded a CT scan and showed an abnormal position of the electrode array, through intraoperative dislodgement. Another surgery performed in a different hospital managed to correct the issue, without the replacement of the device. 

**Fig. 1:**
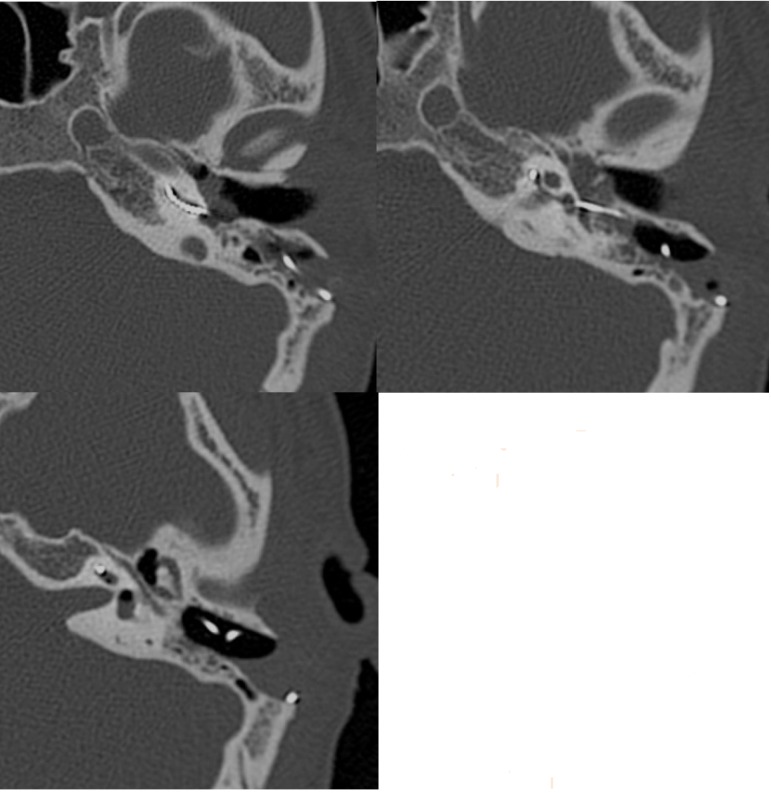
Cochlear implant electrode array surrounded by air, 5 days postoperatively

**Fig. 2:**
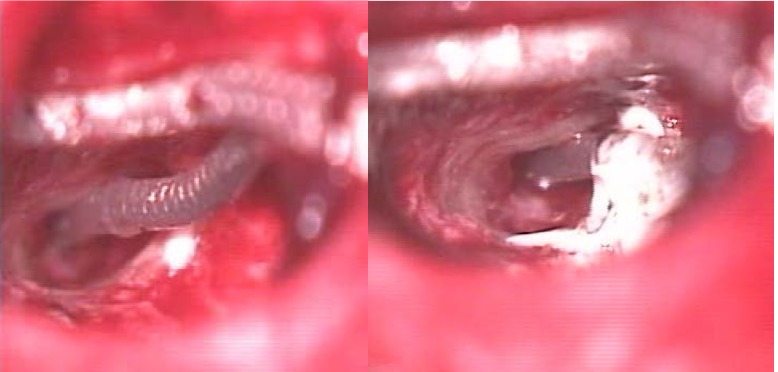
The fixation of the outer part of the electrode array and mastoid bone with glass ionomer cement

The second case in our series occurred at 48 hours postoperatively, also with a straight array and accompanied by a disturbing and persistent vestibular syndrome that failed to alleviate in time. CT scan showed air in the cochlea at 7 days postoperatively (**Fig. 1**). Corrective surgery included electrode array fixation with glass ionomer cement (**Fig. 2**) and the resealing of the cochleostomy with muscle fragments, followed by immediate symptom relief. 

**Fig. 3:**
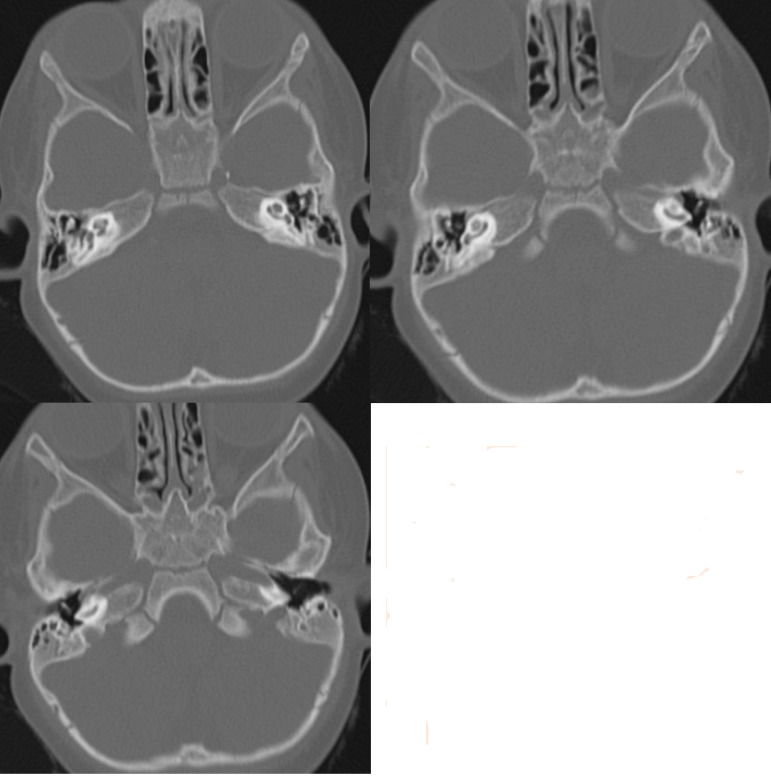
Mastoid CT scan of the patient with meningitis in his history

One case presented for cochlear implanting 1 year after he had meningitis. Although the CT scan and MRI did not consistently show cochlear obliteration (**Fig. 3**), we could not perform a suitable cochleostomy to accommodate a full-length electrode array. We chose not to implant that child, considering that a surgical failure was not covered by the manufacturer of the device.

**Fig. 4:**
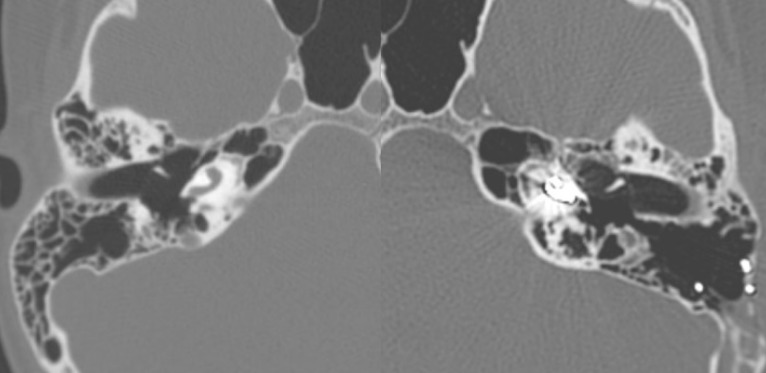
The CT scan of the inner ear in a case having only basal turn of the cochlea (left – preoperatively, right - with folded cochlear implant array inside)

One patient presented in our department with obvious malformation of the cochlea. Cochlear hypoplasia with the presence of only the basal turn was noted on CT scans (**Fig. 4**). Surgery was performed with short electrode array but some difficulties were noted during the procedure. After, the activation of the device, good hearing thresholds were recorded but with poor intelligibility results. Imagistic data confirmed that the inner ear array was folded into the basal turn of the cochlea. Some electrodes were inactivated by software, with better sound perception reported by the patient consequently. He is still on follow-up in the audiology department, for long-term results. 

**Fig. 5:**
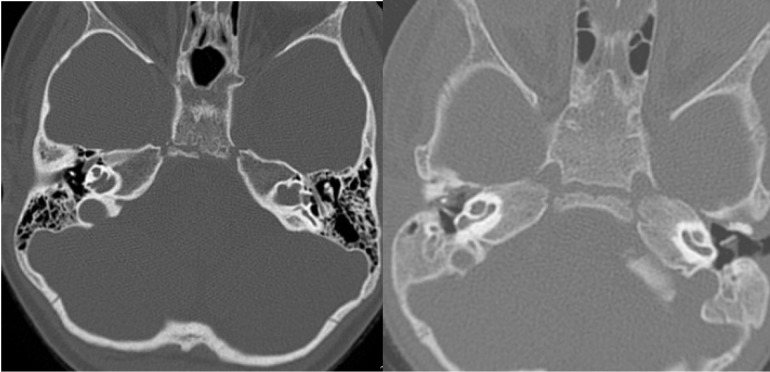
Gusher cases (left – incomplete partition type III; right – apparently normal cochleas)

2 gusher cases were encountered in our cochlear implant patients. One of them was expected preoperatively because of a type III incomplete partition of the cochlea (X-linked deafness). The other one showed no abnormality on imagistics and was an intraoperative surprise (**Fig. 5**). The first patient was solved by means of a tight cochleostomy and thoroughly packing with muscular pieces around the electrode array. No vestibular symptoms were recorded postoperatively. The second one benefited from the “cork style” of the electrode array at its external base, which perfectly occluded the cochleostomy and efficiently prevented any perilymph leak. 

One of our patients reported focal neurological episodes after cochlear implant surgery. Reviewing the technique, it showed a more advanced drilling of the surgical bed for the implant and use of pins for device fixation. Although we did not conduct a team research of the symptom origins, we could hypothesize about the role of trauma surgery in patient’s evolution, considering she had no similar manifestations before surgery. 

## Discussion

Cochlear implantation is a common procedure in medical facilities addressing hearing loss patients. The technique is straightforward today, with few modifications due to relative risks and anatomic limitations of the mastoid and tympanic cavities. Most complications cited in the literature regard flap issues and local infections, with possible viability and extrusion problems. We did not encounter any of those complications. The reasons could be: use of systematic antibiotic prophylaxis postoperatively, meticulous skin flap surgery (2 flaps – cutaneous and muscular, completely closed with non-superimposed sutures), haematoma prevention by tight subcutaneous sutures and possibly the use of minimal access surgery [**12**]. 

**Fig. 6:**
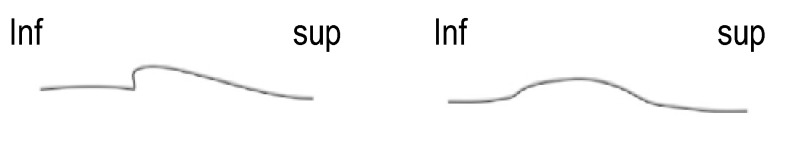
Sharp drilling of the inferior edge of the implant cavity

Migration of the cochlear implant from its bed is possible when no sutures are used to fix it. Drilling a sharp edge of the cavity at its inferior aspect is one way to prevent such a problem (**Fig. 6**). What is worth noting is that in small children, the cranial bone is very thin, making use of transfixion/ fixing sutures difficult to perform. 

The ossification of the cochlea is mentioned in literature as a known major difficulty in patients having meningitis in their medical record. Still, one would expect to discover the process to a certain degree from imagistics. Our case proved that it is not always possible to ascertain these abnormalities in detail. Performing an operation on such patients should always be done with caution and with different types of electrode arrays at hand. Also, certain rules should be discussed with the manufacturers of cochlear implants regarding a proper decision to implant these cases.

Malformations of the inner ear are always difficult tasks for the surgeon. Some of the cases can show gusher intraoperatively and others prevent a full normal insertion of the array. In order to overcome these limitations, paying attention to operative details can be efficient: perform a small cochleostomy, use conical external base arrays and a thorough intraoperative sealing of the inner ear.

Cochleas that prevent full electrode array insertion can be challenging. The use of short arrays and the partial insertions or voluntary inactivation of some of the electrodes postoperatively can all yield good results, from our experience. 

The discussion about the implant bed and the fixation is still on debate. Although, on esthetical grounds, having the smallest profile of the implant remains a goal, small children do not provide enough bone to drill a deep implant bed. If drilling is performed until a much thin osseous island remains, it is possible that a mechanical compression of the dura appears, by consequent fracture of that bone. That particular case and sometimes the fixation pins present on some of the devices can represent causes for neurological abnormal stimulation. Our reported patient did not have any other symptoms, before the cochlear implant.

## Conclusions

The cochlear implant is a very useful procedure in selected patients. Proper indications, good preoperative imagistics, consistent knowledge of the temporal bone anatomy and meticulous surgical technique help preventing complications in these cases. Particular considerations can also be given to electrode arrays shape, consistency and material. Some of them require a systematic fixation for good results and complication prevention. 
